# Transcriptome Analysis Identifies Piwi-Interacting RNAs as Prognostic Markers for Recurrence of Prostate Cancer

**DOI:** 10.3389/fgene.2019.01018

**Published:** 2019-10-22

**Authors:** Yuanli Zuo, Yu Liang, Jiting Zhang, Yingyi Hao, Menglong Li, Zhining Wen, Yun Zhao

**Affiliations:** ^1^Key Laboratory of Bio-Resource and Eco-Environment of Ministry of Education, College of Life Sciences, Sichuan University, Chengdu, China; ^2^College of Chemistry, Sichuan University, Chengdu, China; ^3^Medical Big Data Center, Sichuan University, Chengdu, China

**Keywords:** piRNA, prostate cancer, biomarker, survival analysis, WGCNA

## Abstract

Prostate cancer remains the second leading cause of male cancer death, and there is an unmet need for biomarkers to identify patients with such aggressive disease. Piwi-inteacting RNAs (piRNAs) have been classified as transcriptional and posttranscriptional regulators in somatic cells. In this study, we discovered three piRNAs as novel prognostic markers and their association with prostate cancer biochemical recurrence was confirmed in validation data set. To obtain a better understanding of piRNA expression patterns in prostate cancer and to find gene coexpression with piRNAs, we performed weighted gene coexpression network analysis. Target genes of three piRNAs have also been predicted based on base complementarity and expression correlativity. Functional analysis revealed the relationships between target genes and prostate cancer. Our work also identified differential expression of piRNAs between Gleason stage 3 + 4 and 4 + 3 prostate cancer. Overall, this study may explain the roles and demonstrate the potential clinical utility of piRNAs in prostate cancer in a way.

## Introduction

Prostate cancer is one of the most common male malignant tumors and the second leading cause of cancer death in men worldwide (Mei et al., 2013; Damborský et al., 2017). Currently, the most common clinical diagnostic indices of prostate cancer prognosis include age, prostate-specific antigen level, tumor volume, perineural invasion, and Gleason grading (Carlsson and Roobol, 2017). However, using clinical parameters alone is not sufficient for accurate prognosis (Patel and Gnanapragasam, 2016). Thus, biomarkers that can provide more accurate risk stratification and help clinicians to make improved decision at the pretreatment stage are urgently needed (Long et al., 2014).

Noncoding RNAs (ncRNAs) have been gaining recognition for their involvement in genetic and epigenetic regulation (Cech and Steitz, 2014). Recent studies suggest that ncRNAs could be a promising hallmark in human diseases, particularly in cancer (Esteller, 2011). Studies have documented that expression patterns of some ncRNAs such as lncRNA and miRNA had relationships with clinic situation of prostate cancer (Jin et al., 2011; Ru et al., 2012; Zhu et al., 2014).

As important members in ncRNAs family, Piwi-inteacting RNAs (piRNAs) are ∼26- to 32-nt RNAs whose names derive from their association with the PIWI subfamily of Argonaute proteins. piRNAs are first identified in a genetic screen for mutants affecting asymmetric division of stem cells in the *Drosophila* germline (Aravin et al., 2006; Vagin et al., 2006), and they have also been found expressed in stem and other somatic cells (Juliano et al., 2011). piRNAs are best known for their roles in transposable element repression. But they may additionally regulate gene expression through an miRNA-like base complementary mechanism (Lee et al., 2011; Martinez et al., 2015). Recent studies revealed that expression patterns of piRNAs showed markedly different in human multiple myeloma, breast, lung, gastric, and other cancer tissues compared with their corresponding nontumor tissues (Mei et al., 2013; Li et al., 2015; Moyano and Stefani, 2015; Ng et al., 2016). Hence, more thorough analyses must be conducted before utilizing piRNAs as diagnostic and prognostic markers (Assumpcao et al., 2015; Lim and Kai, 2015).

## Materials and Methods

### RNA-Seq Data Sets and Clinic Data

A total of 106 prostate cancer tissues RNA-seq data generated by the Department of Pathology & Laboratory Medicine, Emory University were obtained from NCBI SRA database (SRP036848). The corresponding clinical information was downloaded from NCBI Gene Expression Omnibus (Series GSE54460) (Long et al., 2014).

### piRNA Expression Analysis

SRR files downloaded from NCBI were converted to FASTQ files using the “fastq-dump” tool in sratoolkit.2.8.1-win64. The unpaired reads were abandoned. Resulting FASTQ files were trimmed using Trimmomatic-0.36 to remove low-quality reads (Bolger et al., 2014). The remaining reads were aligned to human genome hg38 using the Spliced Transcripts Alignment to a Reference (STAR-2.5.2b) software (Dobin et al., 2013). The piRNA reference transcriptome was generated for annotation and quantitation by using the information from the piRNABank database (http://pirnabank.ibab.ac.in/) (Sai Lakshmi and Agrawal, 2008). Expression counts of transcripts were quantitated using HTSeq package. Following TMM normalization, expression values were transformed to count per million mapped reads (CPM) using edgeR (Krishnan et al., 2017); piRNAs with CPM values ≥1 in at least 10% of samples were deemed as expressed and taken into further analyses.

### Survival Analysis

**Figure 1:**
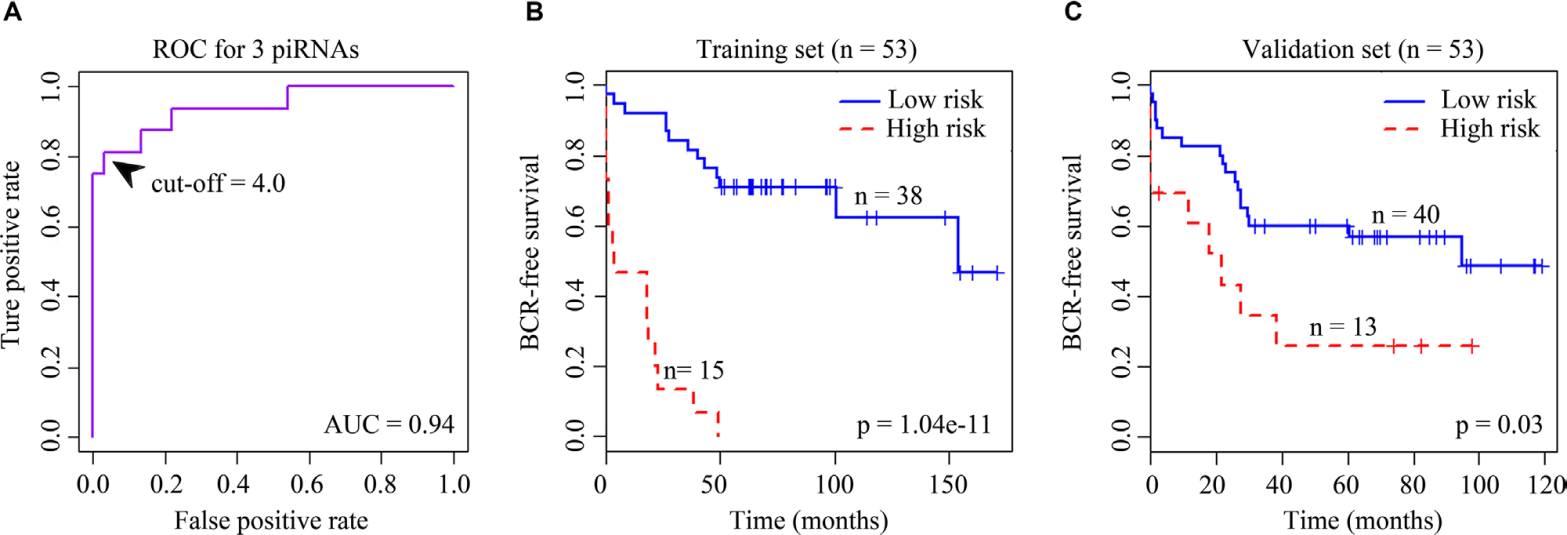
**(A)** Receiver operating characteristic (ROC) curve for the signature of four BCR-associated piRNAs. The ROC curve was generated for BCR predictions with an area under the curve of 0.94. Optimal cutoff value was 4.0. **(B)** Kaplan–Meier survival curves for 53 cases in the training set analyzed by a three-piRNA signature. **(C)** Kaplan–Meier survival curves for 53 cases in the validation set analyzed by a three-piRNA signature.

Clinical information was downloaded from the NCBI GEO database. Patients’ biochemical recurrence (BCR) information was used in survival analysis. We first performed univariate Cox regression analysis to identify candidates significantly associated with patient outcome (*p* < 0.05). Next, a robust likelihood-based survival modeling approach was used to select the piRNA signature. We implemented our analysis by using the “rbsurv” package in R (Cho et al., 2009). Then we built a multivariate Cox regression model by the selected piRNAs to find a final set of piRNAs that had a significant association with BCR of prostate cancer (*p* < 0.01). Both the univariate and multivariate Cox analyses were executed using “coxph” function in “survival” package. Significantly associated piRNAs were used to calculate each patient’s BCR risk. Briefly, we first multiplied a piRNA’s expression value by its corresponding Cox coefficient to obtain an individual piRNA weight. Then we summed all the individual piRNA weights to get the risk score (Firmino et al., 2016; Martinez et al., 2016). And then receiver operating characteristic curve was employed for estimating optimal cutoff points for the outcomes to stratify patients into low- and high-risk groups (Krishnan et al., 2016) (**Figure 1**). The risk score whose corresponding difference between the true-positive rate and the false-positive rate was the maximum was chosen to be the optimal cutoff. Kaplan–Meier curves for two distinct groups of patients were plotted using “survfit” function in “survival” package. *P* value from log-rank test was computed using “survdiff” function.

### Gene Coexpression Network Analysis

**Figure 2:**
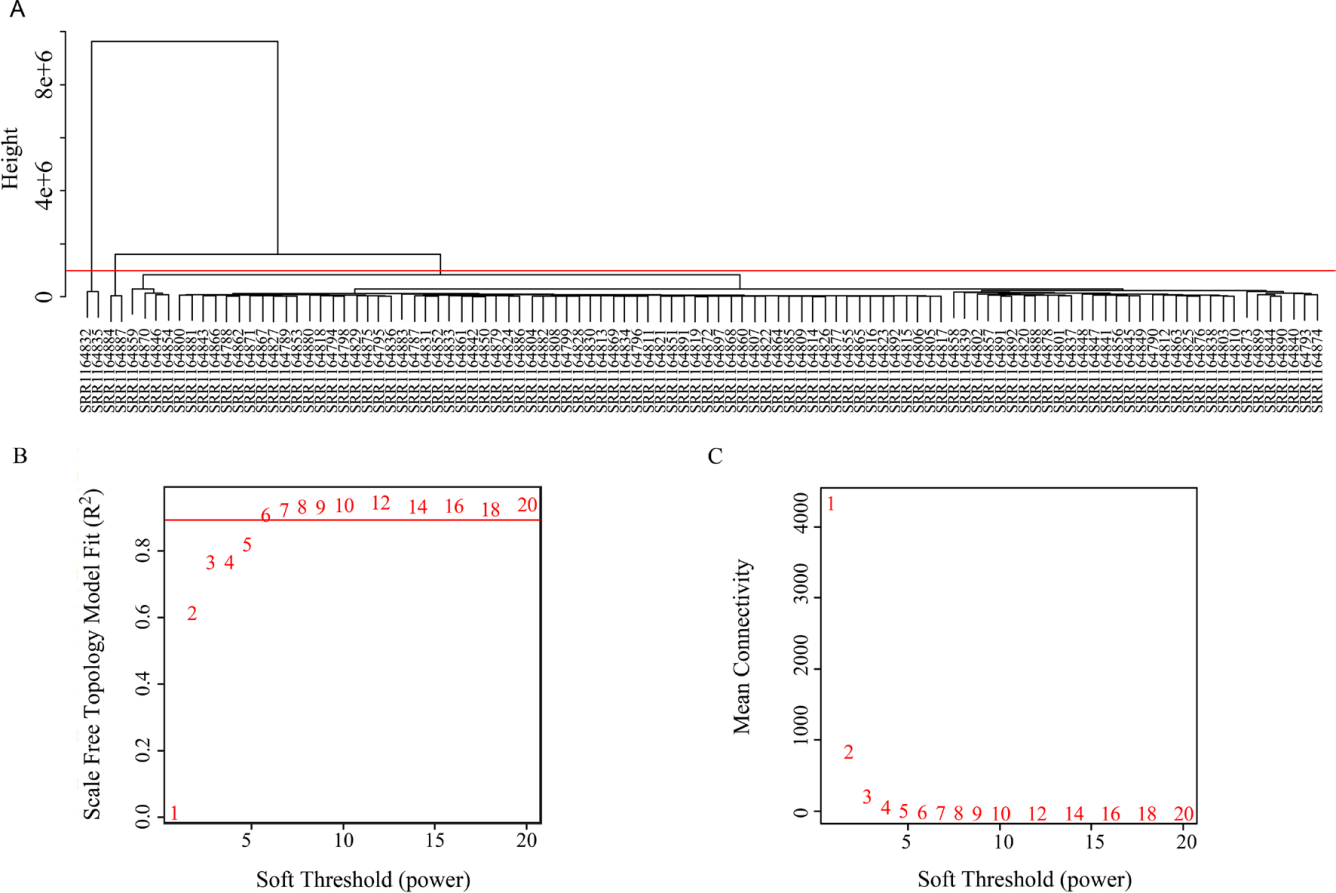
**(A)** Clustering tree of 106 samples. **(B)** Soft threshold corresponding scale independence and **(C)** mean connectivity.

The coexpression analysis was performed using weighted gene coexpression network analysis (WGCNA) method based on the significantly variant genes (SD ≥ 2) and the three survival-associated piRNAs expression data according to the protocols of WGCNA in an R environment (Langfelder and Horvath, 2008). Outlier samples were detected using hierarchical clustering. Setting the cut-height of 1,000,000, we removed four outliers. The remaining 102 samples were taken into the following analysis process (**Figure 2**). We then generated an adjacency matrix by calculating the Pearson correlation between all genes. The PickSoftThreshold function of WGCNA was used to choose the appropriate power for the network topology from various soft thresholding powers. The scale-free network was rendered by raising the soft thresholding power (β) to six, resulting in a scale-free topology index (R^2^) of 0.9 (**Figure 2**) and a mean connectivity approximate of zero (**Figure 2**). The gene coexpression networks were constructed using the blockwiseModules function by a one-step method. Then a topological overlap matrix was calculated using the adjacency matrix, and Interaction networks were constructed for select modules. Cytoscape v 3.5.0 is used for network visualization.

### piRNA Target Prediction

Recent evidence has suggested interaction between piRNAs and mRNAs through base-pair complementarity and a possible inverse correlation between piRNA expression and its corresponding mRNA targets (Hashim et al., 2014; Preethi Krishnan, 2016). But in this study, we did not exclude the possibility that piRNA might have interaction with other RNAs rather than only mRNAs. Fasta sequences of all the genes were obtained from GENCODE database (http://www.gencodegenes.org/releases/26.html) and fasta sequences of the piRNAs were obtained from piRNABank (hg 38). The targets of selected piRNAs were identified using miRanda against the RNA library of human genome with a mean free energy of maximum 20 kcal/mol and alignment score threshold of 140 (Rajan et al., 2016). The resulting RNAs had been taken intersected with the genes that had a coexpression pattern (topological overlap matrix weight ≥0.01) with the piRNAs to obtain the piRNA targets that meet both the requirement of base-pair complementarity (results from MiRanda target predicting) and expression pattern relevance (results from WGCNA coexpression analysis).

### Functional Analysis

Since there was no means to annotate the function of piRNAs directly, we turned to analyze the potential functional insights of piRNAs by focusing on their target genes. Gene Ontology (GO) functional module enrichment, Kyoto Encyclopedia of Genes and Genomes (KEGG) pathway analysis, database searching, and literature consulting were used. GO and KEGG classification of genes targeted by selected piRNA was performed using DAVID functional annotation tool (https://david.ncifcrf.gov/) (Huang et al., 2009). Redundancy in mRNA was removed before analysis. GO terms and KEGG pathways with *p* < 0.05 were taken into consideration to summarize the enriched functions.

### Differential Expression Analysis

Fifty-six Gleason 3 + 4 and 24 Gleason 4 + 3 samples were taken into differential expression analysis. Pairwise comparisons were applied to identify significantly differentially expressed piRNA between the same Gleason stage 3 + 4 and stage 4 + 3 patient cohorts. Differential expression of expressed piRNAs was calculated using DESeq2 version 1.4.1 available in Bioconductor version 2.8. DESeq2 uses a negative binomial distribution model to test for differential expression in deep sequencing data sets. The piRNAs with the absolute value of fold change >1.5 and adjusted *p* value with false discovery rate <0.05 were considered significant.

## Results

### piRNAs Are Associated With Prostate Cancer BCR

We developed a custom analysis pipeline to detect expression patterns of piRNAs in prostate cancer patients from high-throughput sequencing data. There were 7,630 piRNAs expressed with at least 1 CPM in 10% of the samples. Given the limitation of samples, a Holdout method cross-validation was applied to reduce the effect of data variability and avoid overfitting. Briefly, we randomly chose 53 samples as training set and used the rest as validation set.

We first selected an initial set of piRNAs by performing univariate survival analysis using Cox proportional hazards regression model. With the threshold of *p* < 0.05, a total of 808 piRNAs associated with the BCR were initially identified. Next, we screened the optimal survival-associated signature piRNAs based on a robust likelihood-based survival model. Six piRNAs were selected as signature piRNAs that can optimally predict the BCR of patients with prostate cancer. By fitting a multivariate Cox proportional hazards regression model, we finally get a prediction panel that comprised three piRNAs (**Table 1**). The risk scores weighting the BCR of prostate cancer were constructed using the three piRNAs. And then we used a receiver operating characteristic based estimation to get an optimal cutoff score and dichotomized the patients into two groups: low risk (risk score <4.0) and high risk (risk score ≥4.0; **Figure 1**, see *Materials and Methods*). Thirty-eight patients (71.7%) were categorized to the high-risk group, whereas 15 (28.3%) were categorized to the low-risk group. The Kaplan–Meier plot of piRNA risk scores shows that it can distinguish the patients with high risk of BCR from the low-risk patients (log-rank *p* = 1.04e−11, **Figure 1**).

**Table 1:** Three piRNAs significantly associated with BCR of prostate cancer patients.

	Cox coefficient	HR	95% CI	*p*
hsa_piR_005553	0.05357	1.055	1.0305–1.0802	8.14E−06
hsa_piR_019346	0.03015	1.0306	1.0098–1.0519	0.00381
hsa_piR_000627	0.01083	1.0109	1.0032–1.0186	0.00525

### Validation of Three-piRNA Signature

To evaluate the robustness and effectiveness of the three piRNAs signature, we used the rest of the 53 samples as validation set. The BCR risk score of each patient was calculated based on expression values of three piRNAs signature. We further calculated the risk score of each sample and divided the patients into two risk groups based on the Cox coefficients and optimal cutoff risk scores obtained from the training data set.

For the validation data set, 40 (75.5%) and 13 (24.5%) patients were distinguished as the low- and high-risk groups, respectively. Kaplan–Meier plots indicated significant differences between BCRs of the two groups in the validation data set (log-rank *p* = 0.03, **Figure 1**). Similar to the results obtained in the training data set, the risk score showed promising prognostic power of prostate cancer BCR.

### Coexpression Gene Analysis

**Figure 3:**
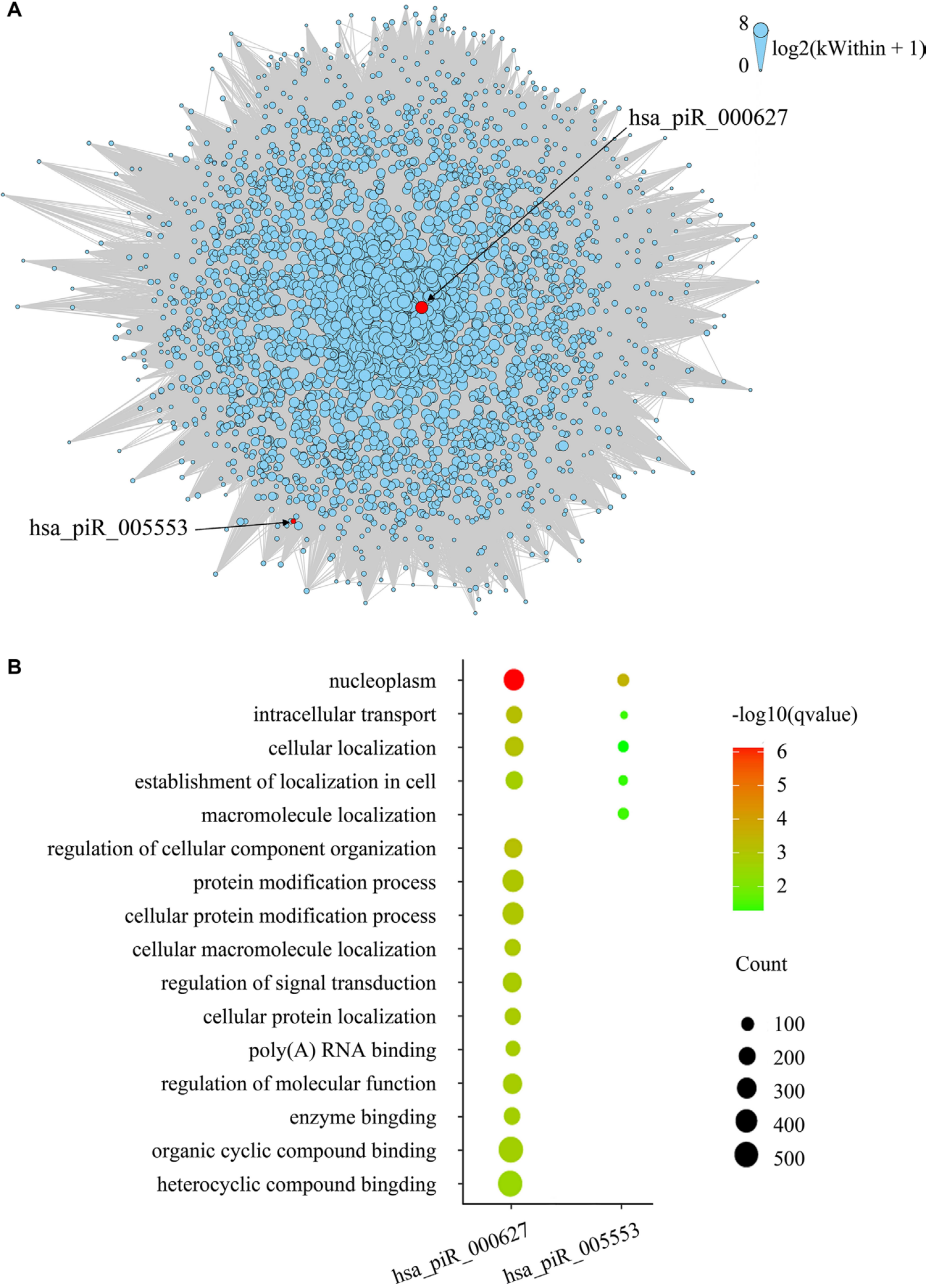
**(A)** Network of the module brown. piRNAs in the modules are represented by the red nodes and labeled. Edges mean the interaction between genes. Intramodule connectivity (*k*_Within_) of each node is represented by the size of node, which was transformed to log2(*k*_Within_ + 1). **(B)** Top gene ontology terms for the targeted genes of hsa_pir_000627 and hsa_pir_005553, respectively. The gene count of each module was represented by the size of spot and the –log10(*q*-value) was represented by the color of the spot.

To evaluate gene expression from a network perspective and gain further insight into the mechanisms by which piRNA changes might influence gene expression, we performed WGCNA to build a gene coexpression network based on the three BCR-associated piRNAs and 37,316 genes whose expression values varied among all the samples (Langfelder and Horvath, 2008). A total of 127 modules were recognized, and two included piRNAs. Module brown consisted of hsa_piR_000627, hsa_piR_005553, and 2,721 other genes (**Supplementary Table 1**), and hsa_piR_019346 had been included in the module lightcyan1 with the other 111 genes (**Supplementary Table 2**). The gene expression networks of the two modules were visualized in Cytoscape. As we can see, hsa_pir_000627 is near the centric position of the network, and hsa_pir_005553 is on the periphery (**Figure 3**). Consistently, the intramodule connectivity (*k*_Within_) of hsa_pir_000627 is 47.46, and hsa_pir_005553 is only 1.71 (median *k*_Within_ value of the whole module is 7.89). It indicates that hsa_pir_000627 might be a hub of this network. It has more coexpression genes and stronger interaction with these genes.

### piRNAs Target Gene Prediction

Recent evidence suggests that piRNAs, in a mechanism similar to miRNAs, may regulate gene expression through base pair complementarity with their targets. However, few studies have identified the corresponding gene targets of specific piRNAs (Hashim et al., 2014; Chu et al., 2015). For this study, we only considered significantly prognosis-related piRNAs (three nonredundant piRNAs in total from BCR) and focused on the correlations between piRNA and its targets. Using MiRanda algorithm v3.3b and applying the cutoffs, we identified nonredundant gene targets of each piRNAs. In order to get targets with more authenticity, we took the intersection of results from MiRanda and the coexpression genes of the three piRNAs. The results are shown in **Table 2** (target genes were listed in **Supplementary Tables 3 and 4**). Intriguingly, we found that 343 target genes (92.45%) of has_pir_005553 were also targets of has_pir_000627. This consists with the results that these two piRNAs are in the same gene module and implied that there might be some interaction between has_pir_000627 and has_pir_005553. GO enrichment also was used for functional analysis of hsa_pir_000627 and hsa_pir_005553 targeting genes (**Figure 3**). As we can see, the GO modules of both hsa_pir_000627 and hsa_pir_007316 had a very high similarity. For instance, both their first modules are nucleoplasm. This might imply their close correlation in biological function furthermore.

**Table 2:** Number of targeted genes of three BCR-associated piRNAs.

piRNA	Targeted gene	Coding gene	Noncoding gene
hsa_pir_000627	1,869	1,556	313
hsa_pir_005553	371	321	50
hsa_pir_019346	1	1	0

Since only one target gene of hsa_pir_019346 was found, we analyzed its function through literature consulting and database searching instead of GO enrichment. The protein coded by the target gene *PNPLA7* (patatin-like phospholipase domain containing 7) is a member of human patatin-like phospholipase domain containing proteins family, which worked as an insulin-regulated lysophospholipase (Kienesberger et al., 2009). Human *PNPLA7* is predominantly expressed in prostate and pancreas; it is involved in regulation of adipocyte differentiation and induced by metabolic stimuli (Wilson et al., 2006). Its related pathways are metabolism and glycerophospholipid biosynthesis. GO annotations related to this gene include lysophospholipase activity and hydrolase activity. Recent work revealed that its gene polymorphism correlated with menstrual disorder. But no work suggests that it is directly associated with human tumor so far.

### Differential Expression of piRNAs Between Gleason Stage 3 + 4 and 4 + 3

Gleason score is known to be a powerful metric that can used to stratify prostate cancer patients into different risk categories. The grading system for prostate cancer is unique in that the final pathologic grade is a Gleason sum of the primary Gleason patterns and the secondary pattern. It has been suggested that primary Gleason 4 pattern and Gleason 3 pattern tumors represent different disease states (Chan et al., 2000; Lavery and Droller, 2012), and several studies suggested that different primary Gleason patterns of patients with a Gleason score of 7 will result in different clinical outcomes (Herman et al., 2001; Berg et al., 2014).

**Figure 4:**
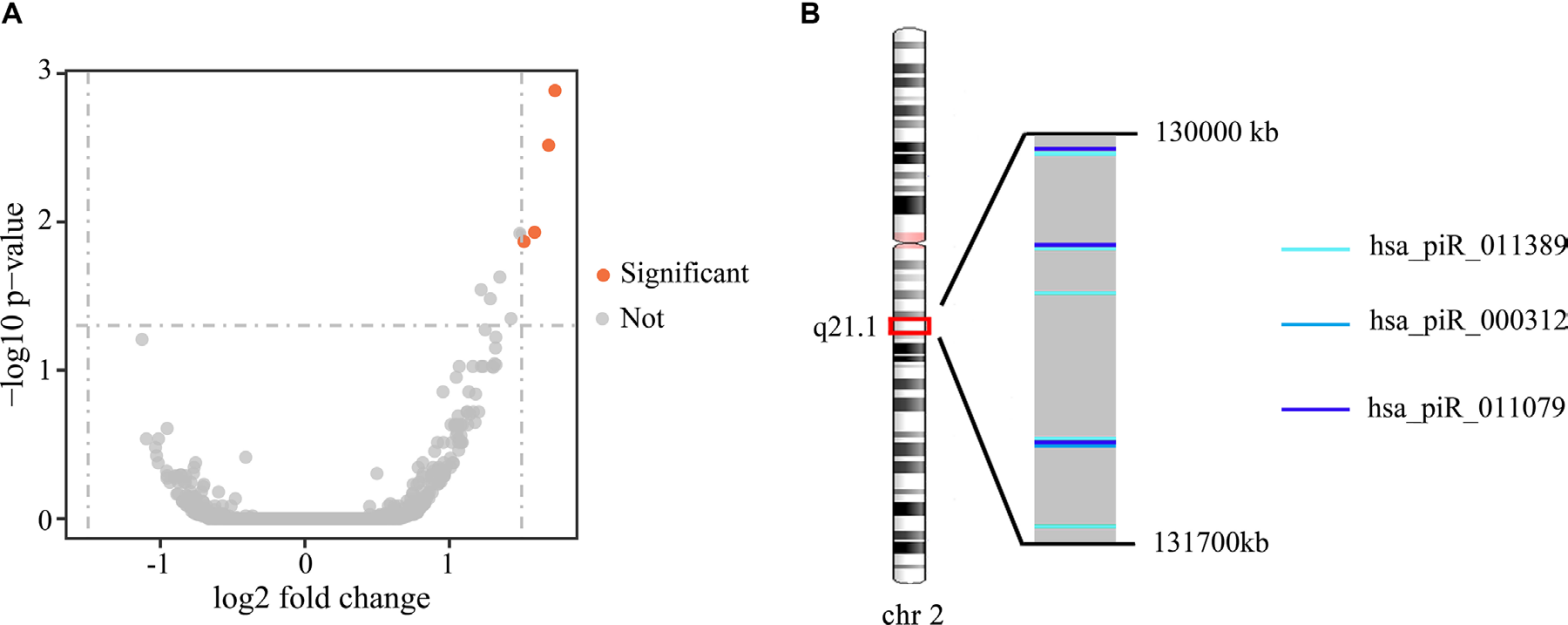
**(A)** Volcano plot of the differentially expressed piRNAs between Gleason 3 + 4 and Gleason 4 + 3 patients. Significant differential piRNAs were represented by the red spots. **(B)** Genomic location of three differentially expressed piRNAs.

To investigate the differences in gene expression, we performed DEseq2 differential expression analysis to explore piRNAs differentially expressed between 56 samples with Gleason 3 + 4 (primary pattern 3) and 24 samples with Gleason 4 + 3 (primary pattern 4). When setting the thresholds that the absolute value of fold change is >1.5 and adjusted *p* < 0.05, we identified four differentially expressed piRNAs (**Table 3**, **Figure 4A**). Interestingly, all the four piRNAs were up-regulated in Gleason 4 + 3 compared with 3 + 4 cases. And we also found that three out of four differentially expressed piRNAs have very close locations in q21.1 of chromosome 2 (**Figure 4B**). This might imply that they came from the same piRNA cluster.

**Table 3:** Four piRNAs differentially expressed between stage 3 + 4 and stage 4 + 3 patients.

	log2FC	*p*	*p*_adj_
hsa_piR_011389	1.730631697	2.70E−07	0.00130497
hsa_piR_000312	1.686705408	1.26E−06	0.003051624
hsa_piR_011079	1.589715335	7.30E−06	0.01174993
hsa_piR_012366	1.516241812	1.69E−05	0.01356796

## Discussion

Prostate cancer remains the most common male cancer, and with over 28,000 deaths per year, it ranks second among tumor mortality (Long et al., 2011; Long et al., 2014). Nowadays, measures for diagnosis and prognosis of prostate cancer have improved a lot. However, major challenges about improvement of prognosis accuracy remain. The inconsistency between results of different methods usually made the physicians and patients disoriented. As a class of important small ncRNA, piRNA gained a growing concern. More and more studies focused on their correlation with human diseases, especially cancer.

In this study, we assessed piRNA expression in 106 prostate cancer samples from NCBI database using a custom piRNA analysis pipeline. Our findings revealed that piRNAs were expressed in human prostate cancer tissues. In addition, we identified three piRNAs (hsa_pir_000627, hsa_pir_005553, hsa_pir_019346) associated with prostate cancer BCR. We also successfully validated the piRNAs’ prognostic significance through cross-validation. Using WGCNA, we constructed the piRNA-correlated gene networks. The results indicated that hsa_pir_000627and hsa_pir_005553 were in a same network module and had a close relation. Gene targets of three candidate piRNAs have also been identified. We found that hsa_pir_000627 and hsa_pir_005553 had 343 cotargeting genes, and they account for 92.45% of targets of has_pir_005553. Functional analysis indicated that both their target genes were mainly associated with nucleoplasm and intracellular transport. The little number of target genes of has_pir_019346 might explain why it had been assigned to a small module. Since its target gene *PNPLA7* is insufficiently studied so far, the biological function and association with human prostate cancer of hsa_pir_019346 needs a further investigation. Moreover, we found four piRNAs differentially expressed between Gleason stage 3 + 4 and 4 + 3 patients. This might be a helpful information to solve the puzzle of accurately distinguishing these two groups.

## Conclusions

In conclusion, our data revealed that three candidate piRNAs, namely, hsa_pir_000627, hsa_pir_005553 and hsa_pir_019346, had significant correlation with BCR of prostate cancer and can be potential prognostic biomarkers. The comparison of Gleason stage 3 + 4 and 4 + 3 cases identified four differentially expressed piRNAs. This shows the utility of piRNAs in clinical classification. In a word, our study shows that piRNAs had potential to be prognosis biomarkers of prostate cancer.

## Author Contributions

ZW designed the experiments. YZu, YL, JZ, and YH performed data analysis. YZu wrote the initial version of manuscript. YZu, YL and ML prepared all the figures. ZW and YZh discussed the results and revised the manuscript. All authors contributed to discussions regarding the results and the manuscript.

## Funding

This project was supported by a grant from the National Natural Science Foundation of China (no. 21575094).

## Conflict of Interest

The authors declare that the research was conducted in the absence of any commercial or financial relationships that could be construed as a potential conflict of interest.
